# A case report of primary cardiac myxofibrosarcoma presenting with severe congestive heart failure

**DOI:** 10.1186/s13019-016-0490-3

**Published:** 2016-07-07

**Authors:** Kosuke Ujihira, Akira Yamada, Naritomo Nishioka, Yutaka Iba, Ryushi Maruyama, Katsuhiko Nakanishi, Ai Shimizu, Kanako C. Hatanaka, Tomoko Mitsuhashi, Toshiya Shinohara, Hatsue Ishibashi Ueda

**Affiliations:** Department of Cardiovascular Surgery, Teine Keijinkai Hospital, 1-12 Maeda Teine-ku, Sapporo, 006-0811 Japan; Department of Surgical Pathology, Hokkaido University Hospital, N14W5 Kita-ku, Sapporo, 060-8648 Japan; Department of Pathology, Teine Keijinkai Hospital, 1-12 Maeda Teine-ku, Sapporo, 006-0811 Japan; Department of Pathology and Biobank, National Cerebral and Cardiovascular Center, 5-7-1 Fujishirodai, Suita, 565-8565 Japan

**Keywords:** Cardiac tumor, Myxofibrosarcoma, Heart failure, Cryoablation

## Abstract

**Background:**

Primary cardiac sarcomas are extremely rare. Furthermore, the myxofibrosarcomas are one of the rarest forms of cardiac sarcomas, and its prognosis is known to be quite poor.

**Case presentation:**

This is a case of a 23-year-old man who presented with acute severe congestive heart failure caused by almost complete obstruction of the mitral valve due to a large left atrial tumor. The patient required endotracheal intubation before his arrival to the hospital, and underwent an emergent surgical excision of the tumor. The tumor had a complex shape and originated from the orifice of the right upper pulmonary vein. Because the tumor seemed to extend over most of the surface of the left atrium, it seemed impossible to reconstruct the left atrium had we done a complete transmural resection. Instead, we carefully peeled the tumor leaving the outer layer of the left atrial wall. We applied cryoablation to the attached site, in order to prevent a recurrence of the tumor. The pathology report revealed that the tumor was a myxofibrosarcoma, and it seemed to originate from the heart. The patient received radiation therapy after the surgery and continues to be alive and well after 1-year, without apparent recurrence.

**Conclusions:**

Cardiac myxofibrosarcoma can cause acute, severe left-sided heart failure. Non-transmural atrial wall resection with cryoablation might be effective for patients with cardiac myxofibrosarcomas with extensive atrial attachment.

## Background

The majority of cardiac tumors are benign, and approximately half of them are myxomas [1]. Primary cardiac sarcomas are rare malignant primary tumors of the heart, accounting for about 20 % of all primary cardiac tumors [2]. Myxofibrosarcomas are reported as one of the rarest forms of cardiac sarcomas [1]. The clinical presentation depends on the site of the tumor and it varies from symptoms of congestive heart failure to thromboembolism and arrhythmias [3–6]. In this report, we present a 23-year-old man who presented with symptoms and signs of acute severe left-sided heart failure, requiring an emergent endotracheal intubation followed by cardiac surgery, caused by a primary cardiac myxofibrosarcoma in the left atrium.

## Case presentation

A 23-year-old man called for an ambulance due to a sudden onset of severe dyspnea. He had 1-year history of general malaise without a major change in his weight or other health problems. At age 13, he had been diagnosed with Asperger syndrome for which he received pharmacological treatment and psychiatric supervision. Otherwise, he was a healthy young man.

**Fig. 1:**
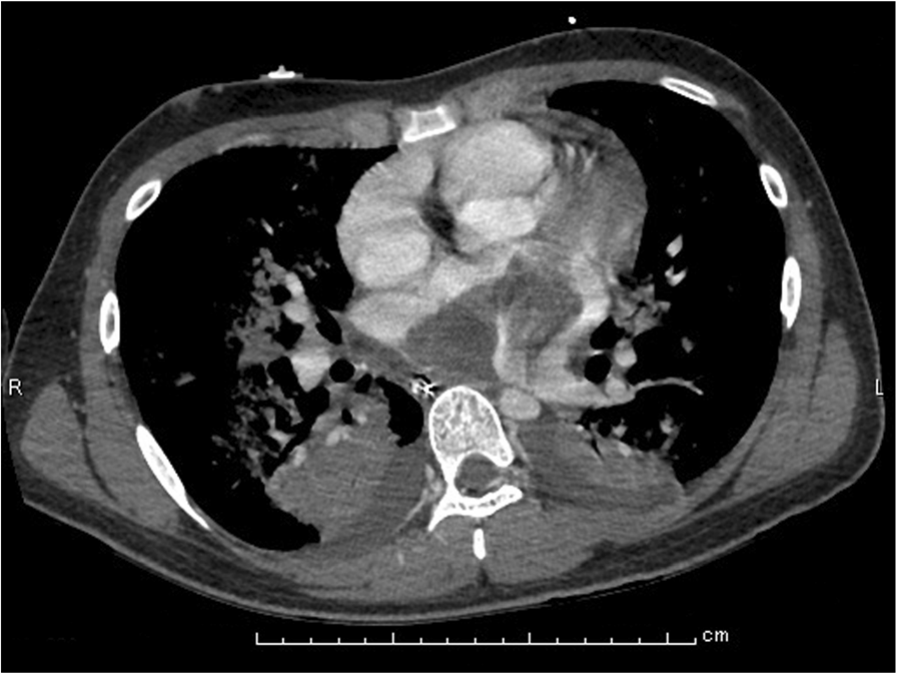
Pre-operative CT image. An enhanced CT scan showed lung congestion and intra-atrial tumor occupying the entire left atrial cavity

At admission, the patient showed clinical signs of acute severe left-sided congestive heart failure; a chest x-ray showed bilateral pleural effusions and a butterfly shadow. A 2-dimensional echocardiogram revealed that an intra-atrial tumor occupied the entire left atrial (LA) cavity and that it had prolapsed across the mitral valve without incarceration. The tumor had both solid and cystic components, and its stalk seemed to be extensively attached to the posterior LA wall. An enhanced computed tomography (CT) scan of the thorax also revealed a similar picture (Fig. 1).

**Fig. 2:**
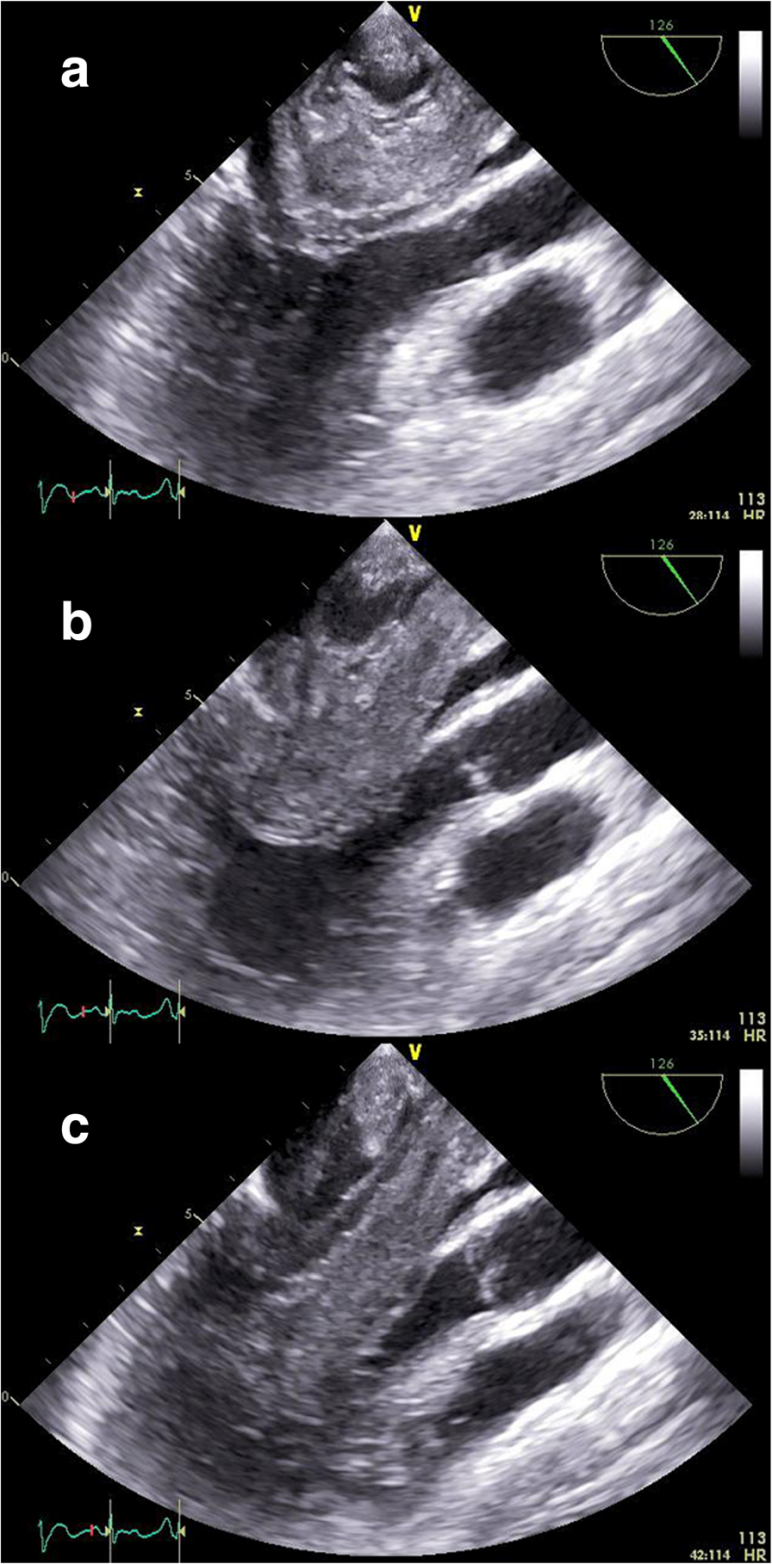
Pre-operative trans-esophageal echocardiogram images. The mobile tumor contracted inside the left atrium during a systolic phrase (**a**) and expanded across the mitral valve (**b**) into the left ventricular during a diastolic phrase (**c**)

The patient underwent an emergent open-heart surgery under cardiopulmonary bypass. The LA was approached through a right-sided left atriotomy. The tumor occupied the entire LA cavity and extended to the entry of the right upper pulmonary vein (PV). Macroscopically, the tumor had three differentiated bodies with a shared stalk. One was a thin and fragile tumor widely attached to the posterior LA wall and extended from the entry of the right upper PV to the edge of the mitral annulus. The others’ stalk originated from this tumor. The second tumor was cyst-like with a smooth surface and it contained a bloody liquid with no thrombi. Its wall was very fragile and it was extracted as small pieces from the LA. We were unable to measure its length and width in vivo, but the pre and intra- operative echocardiography revealed that this balloon-like tumor contracted inside the LA and expanded into the left ventricular lumen through the mitral valve with each heartbeat (Fig. 2). The third one was a solid, club-like tumor. Its stalk was about 1 cm, the head was about 3 cm in diameter, and its length was about 7 cm. This tumor herniated across the mitral valve during the entire cardiac cycle.

**Fig. 3:**
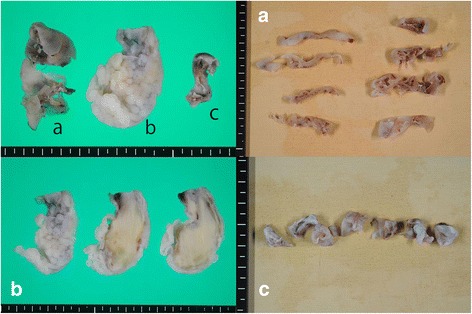
Macroscopic views of the excised tumor. The tumor had three differentiated bodies. **a**, A thin and fragile tumor widely attached to the posterior left arial wall. **b**, A solid and club-like yellow-white colored tumor with myxoid area. **c**, A cyst with smooth surface

These tumors were grossly resected completely from the LA wall (Fig. 3). The thin tumor was easily peeled off from the intima of the LA wall without apparent adhesion between the tumor and the outer layer of the LA wall except the stalk mentioned above. The stalk, which was about 2 cm in diameter and attached to the outer layer of the LA wall, was peeled off leaving the outer layer. Here, we intentionally avoided resecting all the layers of the LA wall because the thin tumor seemed to extend over most of the surface of the LA wall. We felt we would be unable to reconstruct the LA if we attempted to completely resect the tumor and the wall. The rest of the tumors were easily resected with the common stalk. In order to prevent a recurrence of the tumor, we applied cryoablation to the attached site.

After the tumor resection, we identified a mitral valve annular enlargement with severe regurgitation, which was probably caused by the prolapse of the large tumor across the valve. We performed a mitral valve annuloplasty with a Physio II ring (Edwards Lifesciences Corp. Irvine, CA, USA) with satisfactory results. The surgery ended safely without major complications or need for transfusions.

**Fig. 4:**
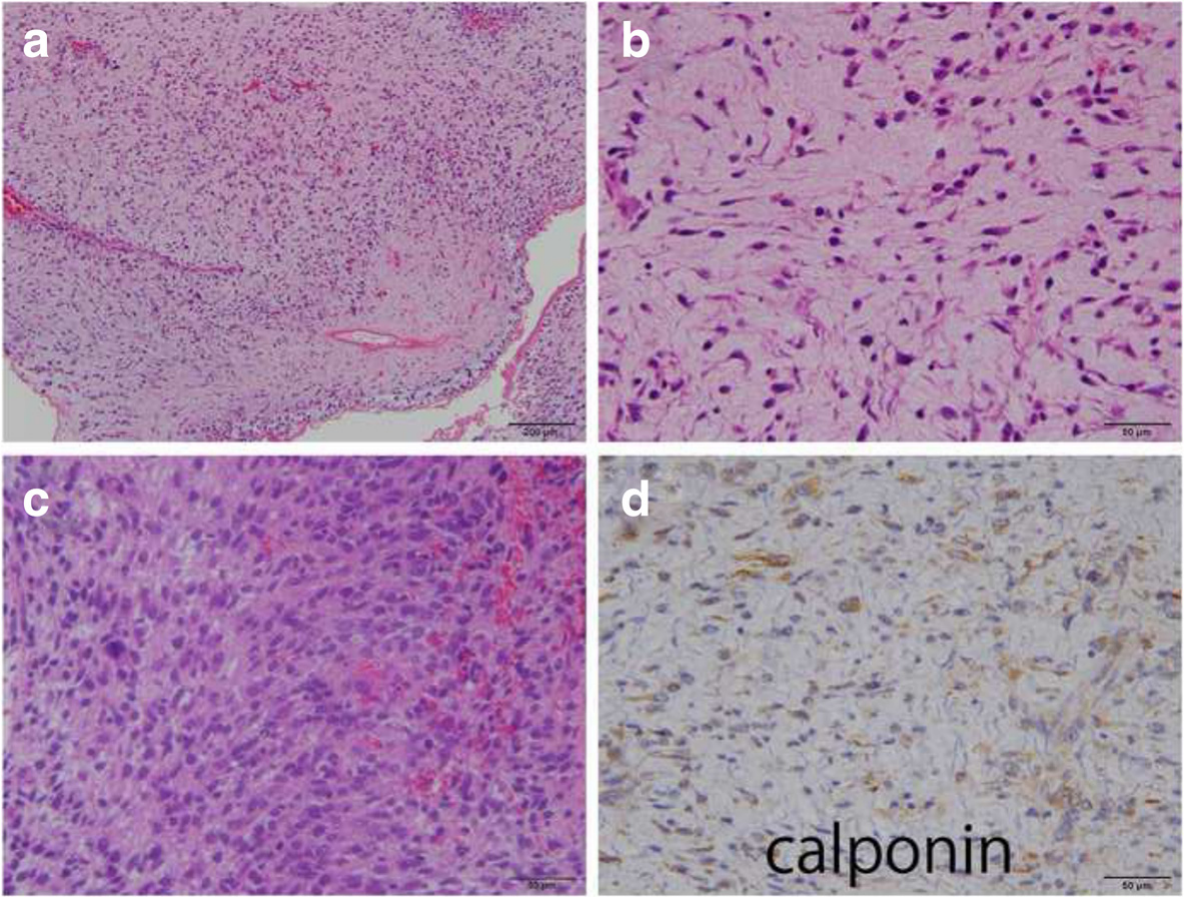
Histological images. **a**, An examination with hematoxylin and eosin technique showed spindle-shaped cells with myxoid background. **b**, Most of the tumor cells showed spindle-shaped, but some cells were round in shape. **c**, Focally a hypercellular lesion was found. **d**, The tumor cells were weakly immunnoreactive for calponin

The traditional histological examination with hematoxylin and eosin technique revealed a tumor composed of spindle-shaped cells with a predominantly myxoid background (Fig. 4). Some tumor cells had round-shapes. Most of the tumor showed hypo to intermediate cellularity but focally the tumor contained a hypercellular area. Mitotic figures were found (4 or less mitoses per 50 high-power fields) and there was no necrosis.

Immunohistochemical tests showed a weak immunoreactivity for calponin, and patchy positivity for CD34. It was negative for CD31, Factor VIII, desmin, myogenin, MyoD1, h-caldesmon, S-100 protein, CAM5.2 and AE1/AE3. The tumor cells showed focal myofibroblastic differentiation. Therefore, the final diagnosis was myxofibrosarcoma. The possibilites of angiosarcoma or leiomyosarcoma were ruled-out by the histological and immunohistochemical features.

The patient was discharged on post-operative day 10 without complications. A magnetic resonance imaging (MRI) of the brain and an enhanced whole body CT were negative for metastatic disease.

Upon discharge, the patient was referred to a local cancer center where he later received radiotherapy for 2 weeks, as adjuvant therapy. His postoperative treatment was completed successfully, and 1-year later he remains asymptomatic without apparent recurrence. A transthoracic echocardiogram 14 months after the surgery was normal.

## Conclusion

A left atrial sarcoma is an extremely rare cardiac tumor, generally with poor prognosis [7]. One-quarter of all primary cardiac tumors are malignant; approximately 95 % are sarcomas and 5 % are lymphomas. Sarcomas of the heart usually occur in the 3^rd^ or 5^th^ decade of life, and are seen with equal frequency in males and females [8].

A myxofibrosarcoma is a tumor of mesenchymal origin that occurs most commonly in the extremities of elderly patients [9]. Primary myxofibrosarcomas rarely arise in the heart, and they may involve any of the four cardiac chambers, but it most frequently affects the left atrium [10]. Because the primary myxofibrosarcoma is capable of local invasion or distant metastasis, the prognosis of patients has been reported to be very poor despite surgical resection, radiation therapy, and chemotherapy [10].

Cardiac tumors have a variable clinical presentation that includes obstruction to blood flow, embolism and local invasion, resulting in pericardial effusion, arrhythmias or systemic symptoms [3].

A cardiac sarcoma located in the left atrium can cause obstruction of the mitral valve and cause symptoms of mitral stenosis such as dyspnea, cough, hemoptysis, and fatigue, all of which have been reported previously [4]. When the tumors are suspected to incarcerate in the mitral orifice, they require emergent surgery [11]. To the best of our knowledge, there are no previous reports of severe acute congestive heart failure caused by a myxofibrosarcoma, requiring an emergent endotracheal intubation and operation.

Sarcomas are aggressive tumors with an average survival of approximately 11 months. The most important factor that predicts a better prognosis is the ability to achieve a complete surgical resection of the tumor. Life expectancy is nearly twice as long for patients in whom complete tumor resection is achieved, compared with patients who undergo incomplete excision [4]. Hence, surgery is the mainstay of treatment for these lesions, and to date, the extent to which adjuvant chemotherapy and radiation therapy are beneficial, remains controversial. Burke et al. [12] reported 75 cases of primary sarcomas of the heart with better survival rates in the patients who received chemotherapy and radiotherapy. Repeat surgical interventions are reported to be effective, with the patient maintaining a good quality of life between surgical events [5].

Complete resection of the malignant tumor is ideal, however, large tumors with a broad stalk attached to the atrium could make the reconstruction of the atrial wall difficult [5]. Unfortunately, our patient had a quite broad tumor stalk, which extended over most of the left atrial surface. Because of the severity of his presentation, we were unable to perform an extensive preoperative examination or planning. It was also mandatory to finish the surgery as soon as possible to decrease his surgical stress. Therefore, we performed a non-transmural atrial wall resection leaving pericardium, with cryoablation on its stalk near the right upper pulmonary vein. This operation might be useful for intimal cardiac tumors only and should not be performed for tumors arising from the pericardium including metastasis. Fortunately in this case, the pathology revealed that the tumor was not a metastasis, so we decided not to add a secondary surgical intervention in the acute post-operative phase. Due to the possibility of incomplete resection of the tumor, we recommended adjuvant radiation therapy along with follow-up echocardiograms every 3 months.

In summary, primary cardiac sarcomas are rare malignant tumors of the heart with poor prognosis. Clinical features depend on the site of the tumor and may include severe acute left sided heart failure for which emergent tracheal intubation is necessary. Complete surgical resection provides the greatest chance for survival; however, non-transmural atrial wall resection with cryoablation might be effective for patients with extensive atrial attachment. Careful follow-up is necessary to detect recurrences.

## Abbreviations

CT, computed tomography; LA, left atrial; PV, pulmonary vein
